# Drivers’ Visual Search Patterns during Overtaking Maneuvers on Freeway

**DOI:** 10.3390/ijerph13111159

**Published:** 2016-11-19

**Authors:** Wenhui Zhang, Jing Dai, Yulong Pei, Penghui Li, Ying Yan, Xinqiang Chen

**Affiliations:** 1 Traffic School, Northeast Forestry University, Harbin 150040, China; andychen166168@163.com (J.D.); peiyulong@nefu.edu.cn (Y.P.); 2 State Key Laboratory of Automotive Safety and Energy, Tsinghua University, Beijing 100084, China; liph2013@163.com; 3 Department of Automobile, Chang’an University, Xi’an 710064, China; yanying2199@chd.edu.cn; 4 Merchant Marine College, Shanghai Maritime University, Shanghai 201306, China; chenxinqiang2005@163.com

**Keywords:** visual behavior, overtaking, freeway, driving simulator

## Abstract

Drivers gather traffic information primarily by means of their vision. Especially during complicated maneuvers, such as overtaking, they need to perceive a variety of characteristics including the lateral and longitudinal distances with other vehicles, the speed of others vehicles, lane occupancy, and so on, to avoid crashes. The primary object of this study is to examine the appropriate visual search patterns during overtaking maneuvers on freeways. We designed a series of driving simulating experiments in which the type and speed of the leading vehicle were considered as two influential factors. One hundred and forty participants took part in the study. The participants overtook the leading vehicles just like they would usually do so, and their eye movements were collected by use of the Eye Tracker. The results show that participants’ gaze durations and saccade durations followed normal distribution patterns and that saccade angles followed a log-normal distribution pattern. It was observed that the type of leading vehicle significantly impacted the drivers’ gaze duration and gaze frequency. As the speed of a leading vehicle increased, subjects’ saccade durations became longer and saccade angles became larger. In addition, the initial and destination lanes were found to be key areas with the highest visual allocating proportion, accounting for more than 65% of total visual allocation. Subjects tended to more frequently shift their viewpoints between the initial lane and destination lane in order to search for crucial traffic information. However, they seldom directly shifted their viewpoints between the two wing mirrors.

## 1. Introduction 

Overtaking is one of the most common events observed on freeways. When the speed of a leading vehicle is lower than expected, the following vehicle will accelerate and overtake it [1]. A sequence of overtaking typically consists of three phases, namely lane-changing, accelerating, and lane-returning. Firstly, the driver in a following vehicle ought to observe traffic conditions in the destination lane by use of the left wing mirror, and search for an appropriate opportunity to change lanes. In the second phase, the driver should not only successfully capture the motion of the leading vehicle in order to keep a safe horizontal space, but also constantly observe the longitudinal traffic conditions in order to keep a safe headway. Before returning to the initial lane, the driver will also estimate headways with other vehicles by use of the right wing mirror. Although overtaking lasts only several seconds, drivers should have a prior or concurrent observation, analysis, and judgment in order to make successful decisions [2].

As overtaking involves abrupt and short-term visual searching and decision making, it may easily result to an increase in the accident risk [3]. According to the statistics provided by the Ministry of Public Security of China, overtaking accidents accounted for nearly 30% of freeway accidents from 2010 to 2015. Moreover, the primary causation of these accidents was attributed to drivers’ insufficient observation which caused wrong decision making (e.g., improper overtaking opportunities and faulty maneuvers). Therefore, visual searching strategies during overtaking are associated with accidents. This is especially true for novice drivers, whose visual search patterns differ from experienced drivers. Experienced drivers tend to allocate their viewpoints more widely in the horizontal plane and farther in the longitudinal direction, however, novice drivers tend to pay more attention to the more narrow scope in front of them. It is of importance to improve their capabilities to skillfully detect potential hazards [4,5,6].

For drivers’ eye movements, most researchers focus on blinking, gazing, and scanning. Researchers typically use blinking frequency to evaluate driving fatigue and workloads [7,8,9,10]; gazing and scanning, however, are only considered as effective means to gather traffic information and recognize drivers’ intentions [11,12,13,14,15,16,17]. Common analysis metrics include gaze duration, gaze frequency, saccade duration, saccade frequency, saccade amplitude, and various transition-based parameters between fixation. Actually, little visual processing can be achieved during a saccade. However, a saccade can reposition drivers’ viewpoints from one region to another. Thus, we primarily collected gazing and scanning data of subjects in this study. In past years, most researchers applied driving simulators to study drivers’ visual modes because driving simulation experiments have been safe and reliable under some hazardous conditions. As such, this study employs a driving simulator to analyze gazing and scanning modes of subjects during overtaking maneuvers and presents the proper attention allocating modes between areas of interest (AOI). 

The rest of this paper is organized as follows. Section 2 involves an extensive literature review about relationships between visual behaviors and safety. Section 3 describes the detailed experimental designs and data collections. Section 4 characterizes gaze and saccade behaviors of subjects. Section 5 gives a brief discussion of relevant issues. Section 6 draws some conclusions and presents further research.

## 2. Literature Review

### 2.1. Overtaking and Safety

Drivers typically implement overtaking maneuvers according to current speed, headway between vehicles, traffic flow, and traffic infrastructures. Therefore, they need to collect and deal with traffic information, correctly judge potential opportunities, detect potential collision risks, and resolve conflict during an overtaking process. The possibility of misjudgment tends to be high during this complicated process. Overtaking maneuvers on freeways, while only lasting a few seconds, presents drivers with risk of lateral and rear-end crashes [18,19]. Thus, when making such movements, drivers need to pay more attention to the essential areas in their fields of vision due to these aforementioned potential conflicts. Until now, research on overtaking maneuvers has focused on modeling the sight distance [20], the lateral displacement [21], the speeds of both following and leading vehicles [22], and the gap acceptance of impatient drivers [23]. The researchers have generally employed driving simulators and field tests to collect traffic data, then developed models. These models have been quite popular in guiding driving behaviors and designing auxiliary safety devices. 

Moreover, some studies have focused on overtaking maneuvers including drivers’ decision making models [19] and operations [24,25]. These research findings contribute to provide valuable guidance for drivers’ performances during overtaking maneuvers. For example, the minimum safe distance, lane-change model, and other collision warning models have been applied in the field of active safety. In addition, the study of vehicles’ trajectory during overtaking, following the shape of a sine-wave [26], helps to control the overtaking vehicle more precisely.

### 2.2. Visual Attention and Safety

Eye movements have become one of the hotspots in the field of driving behavior. A drivers’ activity can be recognized based on eye and head tracking data. The basic eye patterns, namely saccades, fixations, and blinks, have been first considered as a possible information source for risk detection. The related research findings have demonstrated that the higher accident rate of novice drivers is due to their lack of driving experiences. Different visual searching modes and visual field loss influence driving performances, such as lane keeping and gap judgment [27,28,29,30,31,32]. Therefore, visual attention has been considered to be one of the primary contributing factors for accidents. Underwood et al. [33,34,35,36] conducted systemic experiments to examine the influences of driving experiences and traffic environments to drivers’ visual attention. They found that experienced drivers more widely and flexibly allocated their vision during complicated driving tasks than novice drivers. In addition, experienced drivers fixed more on wing mirrors and poor visibility conditions that decreased their visual searching ability [12,37,38]. Young drivers’ inattention, including drowsiness and distraction, was considered to be closely related with traffic crashes [39,40]. Eye movements and visual attention were thus linked in most instances [41].

A substantial number of studies have examined drivers’ eye movements and their references to visual search strategies. The findings have shown that the broader visual distribution along the horizontal axis and shorter time of fixations can help drivers detect potential hazards [42,43]. Traffic hazards tend to arise with the visual field defects and delayed perception [44,45]. In addition, it is suggested that drivers regularly use wing mirrors and the rear view mirror to monitor rearward road conditions [46,47]. In order to improve drivers’ visual scanning abilities, some researchers have recommended training interventions [48,49]. After guidance procedures, drivers showed broader scanning patters in a hazard perception test compared to an untrained control group. Additionally, digital image displays may be applied to present a wider field of view and eliminate blind spots [50].

Despite extensive research in the field of eye movements, so far, there are still some areas lacking in information regarding visual search patterns during overtaking. Normally, drivers tend to look straight ahead and focus on the position where vehicles will reach. When overtaking, however, drivers should allocate their viewpoints differently. This study focuses on presenting an appropriate visual search strategy during overtaking maneuvers which may be applied to correct some drivers’ visual behavior and improve traffic safety. 

## 3. Methods

### 3.1. Apparatus

**Figure 1:**
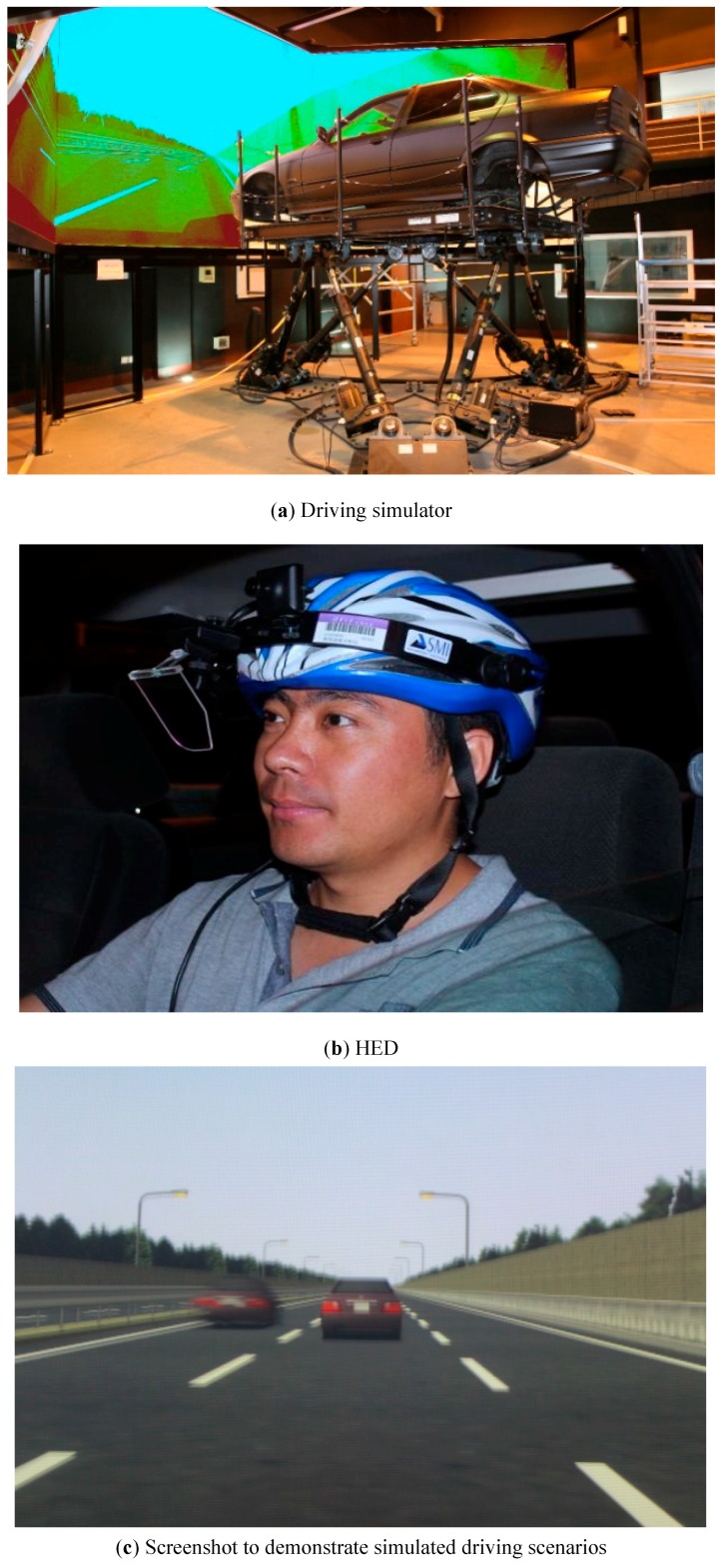
Apparatus and screenshots used during the study. (**a**) Driving simulator; (**b**) SMI iView X HED 4 system (HED); (**c**) Screenshot to demonstrate simulated driving scenarios.

Figure 1a shows the driving simulator used in this study; it consists of a scenario system, vehicle motion system, sound system, vehicle dynamic feedback system, and central control platform. The simulator uses a modified passenger car, the body of which is supported by hydraulic cylinders to allow six degrees of freedom.

SMI iView X HED 4 system (HED) can record data of eye movements with a sampling rate of 50 Hz, as shown in Figure 1b. Two cameras mounted on the helmet can capture drivers’ eye movements and the front traffic video individually. Behavioral and Gaze Analysis 2.5 system (BeGaze) (SensoMotoric Instruments: Berlin, Germany) can analyze the video of eye movements and display visual research modes. The gaze position accuracy of the system is 1°.

Three screens affording a 270° viewing angle can project the virtual driving scenarios in front of the cab, as shown in Figure 1c. Another two screens positioned behind the cab enable drivers to observe the rear traffic information by scanning the rear view mirror or the wing mirrors. The projector has a resolution of 1280 × 768 pixels and a frame rate of 60 Hz.

### 3.2. Participants

One hundred and forty volunteers participated in this study, composing 113 male and 27 female drivers. All participants had held valid driving licenses for more than two years and had driven total distances of more than 20,000 km. Table 1 shows the descriptive statistics of the participants’ sample data. Participants responded to advertisements online and were reimbursed with ¥200 (RMB) as compensation for about 40 min. Furthermore, all participants had normal (1.0 or more) or corrected vision.

### 3.3. Scenario

Two types of traffic scenarios, consisting of a freeway with three lanes per direction, traffic flow, traffic signs, buildings, guardrails and trees, were designed. Driving conditions incorporated other road users moving on the road and obeying local traffic laws. We did not design any hazards along the route in order to focus on visibility issues during typical overtaking maneuvers. 

The first scenario was for practicing in traffic conditions that were similar to the real world. Participants drove for about 5 to 20 min to familiarize themselves with the simulator and virtual traffic conditions. Different from the practice scenario, the second scenario was for overtaking experiments. A truck (6915 mm length × 2150 mm width × 2260 mm height) and a car (4523 mm length × 1775 mm width × 1467 mm height) both served, individually, as leading vehicles. The leading speed of the truck or car was 60, 70, 80, 90, and 100 km/h respectively. 

### 3.4. Experiment Design

In this study a leading vehicle operated under five different levels of speed and two different types, considered as two independent variables. Dependent variables were participants’ gaze and saccade parameters. Participants were randomly divided into 10 (i.e., 5 × 2) groups accordingly. Each group has 14 participants, and there were no significant differences in age or driving experiences between the two groups. For comparative purpose, the scenario in each experiment had the same traffic environment.

First, participants followed the leading vehicle at a comfortable and safe distance in the same lane. Here, the comfortable and safe distance refers to the headway distance between the leading vehicle and the simulator at which the participants could operate freely and safely. We designed a visual indication of overtaking on the screen. While seeing the indication, participants would then change lanes, accelerate, pass the leading vehicle, and return to the initial lane at last. 

### 3.5. Procedure

Before the experiments, all participants completed a questionnaire about their age, licensing duration, and driving mileage. After an introduction to the driving simulator, participants wore the HED and began with the practice scenario. Their eye movements were calibrated by use of a five point screen. Overtaking experiments would not start until participants were able to skillfully operate the driving simulator. Although the traffic circumstance was virtual, participants were to obey traffic laws, as they do in daily driving.

The leading vehicle kept static at the beginning of overtaking experiments. When participants were ready for driving, the leading vehicle started to travel at the scheduled speed. Once the overtaking indication appeared on the screen, the participant would search for a good opportunity to overtake the leading vehicle. The HED would track and record their eye movements during the whole process. The overtaking indication was a flashing red arrow on the screen. It would not appear until a subject drove for about 10 min. 

### 3.6. Data Analysis

**Figure 2:**
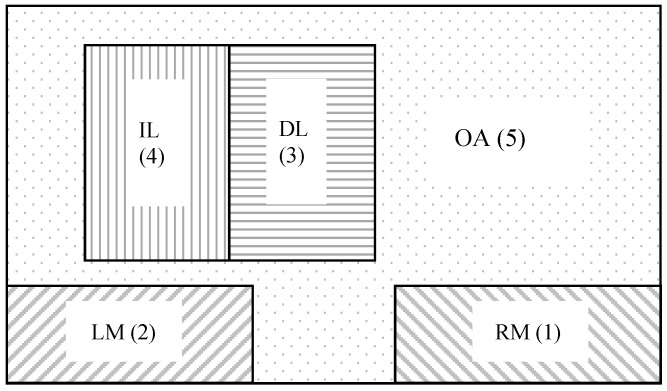
Areas of interest (AOI) during overtaking.

In order to identify key areas that attracted participants’ attention during overtaking maneuvers, AOIs were divided into five sub-regions (i.e., right mirror (RM), left mirror (LM), destination lane (DL), initial lane (IL), and other area (OA), as is shown in Figure 2). This design was based on a previous work conducted by Panos Konstantopoulos et al. [38] which analyzed vertical and horizontal spread of viewpoints. Readers are reminded that in the simulator used in this study, drivers sat on the left side of the cab and vehicles ran on the right side of the road. 

In addition, the raw visual data exported by the eye-tracking system did not conduct data quality control. There was some missing scene information due to errors in pupil detection. The information used in this study only included the coordinates of the gazing points in the scene image.

The HED would record participants’ eye movements when the leading vehicle started, until participants finished the experiment. The portion of the data that was of interest for this study consisted of what was captured between the appearance of overtaking signals and the completion of overtaking maneuvers. BeGaze contributed to the analysis of the video of the eye movements and output the gaze duration and frequency, saccade duration, frequency, and angle.

## 4. Results

### 4.1. Gaze Behavior

During a fixation, the visual points of subjects would spread a certain small field, rather than fix on a position. This characterization required researchers to use a circular field to identify a fixation point. We set a 100 px scope as the small visual field in BeGaze in order to count the number of fixations. BeGaze would identify the gaze behaviors for when pupils stayed over 100 ms within a 100 px scope. The mean gaze duration and gaze frequency are shown in Table 2.

#### 4.1.1. Gaze Duration

**Figure 3:**
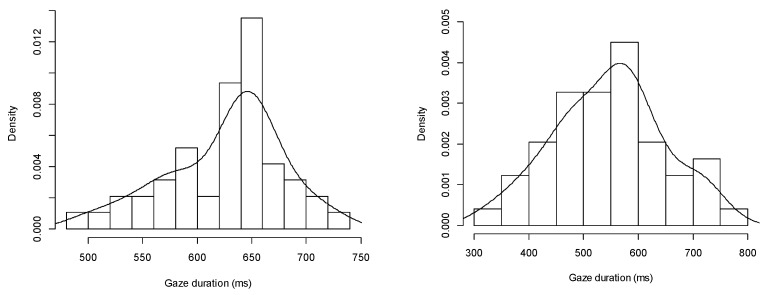
Distribution of a subject’s gaze durations (**a**) when the leading vehicle was a truck; (**b**) when the leading vehicle was a car.

We selected a subject’s visual data at 60 km/h to examine the distribution of gaze duration, as is shown in Figure 3. The gaze durations roughly follow a normal distribution pattern whether the leading vehicle was a car or truck (truck: *p* = 0.086 > 0.05; car: *p* = 0.9039 > 0.05). We examined the gaze duration data at other speed levels, and the same results were drawn.

From Table 2, we can see that as the speed of the leading vehicle increases, the mean gaze duration decreases slightly. However, analysis of variance (ANOVA) shows that no significant difference exited between speed and gaze duration (truck: *F* (4, 65) = 0.2679, *p* = 0.8980; car: *F* (4, 65) = 0.0587, *p* = 0.9935).

At each speed level, the mean gaze duration for the truck was longer than that for the car (more than 60 ms). ANOVA shows that the type of the leading vehicle significantly affected gaze duration at the speed of 60 km/h (*F* (1, 22) = 8.2564, *p* = 0.0123). At other speed levels, we can draw the same results. The gaze duration is thus significantly dependent on the type of the leading vehicle.

#### 4.1.2. Gaze Frequency

The speed of a leading vehicle has no significant impact on gaze frequency (truck: *F* (4, 65) = 0.0862, *p* = 0.3637; car: *F* (4, 65) = 0.5823, *p* = 0.9588). However, the gaze frequency for the truck and the car was about 1.2 Hz and 1.4 Hz, respectively, which shows a significant difference in the ANOVA at each speed level (*p* < 0.05). Thus, similar to gaze duration, gaze frequency was also dependent on the type of the leading vehicle.

### 4.2. Saccade Behavior

Participants shifted their attention from one AOI to another through the saccade behavior. Table 3 shows the observed saccade duration, frequency, and angle. We conducted ANOVA for saccade duration, saccade frequency, and saccade angle. 

#### 4.2.1. Saccade Duration

Saccade durations ranged between 30 ms and 150 ms. Distribution tests indicated that the saccade duration follows a normal distribution pattern. It can be seen from Table 3 that the mean saccade duration increased as the speed of the leading vehicle increased. For example, when participants overtook a truck, the mean saccade duration was only 46.21 ms at 60 km/h, while it nearly doubled to 102.51 ms as the speed increased to 100 km/h. The same conclusion can be drawn when a passenger car served as the leading vehicle. The speed of the leading vehicle thus significantly impacted the saccade duration (truck: *p* = 0.0007 < 0.05; car: *p* = 0.0292 < 0.05). At each speed level, the saccade duration was always a little longer for the truck than for the car. However, no significant correlation exists between the type of the leading vehicle and the mean saccade duration (*p* > 0.05). 

#### 4.2.2. Saccade Frequency

Participants’ saccade frequencies were about 1.0 Hz and 1.2 Hz for the truck and the car, respectively, at each speed level. However, no significant difference can be seen between the type of the leading vehicle (*p* > 0.05). Moreover, there was no significant correlation between the speed of the leading vehicle and the saccade frequency (truck: *p* = 0.4415 > 0.05; car: *p* = 0.8043 > 0.05). 

#### 4.2.3. Saccade Angle

**Figure 4:**
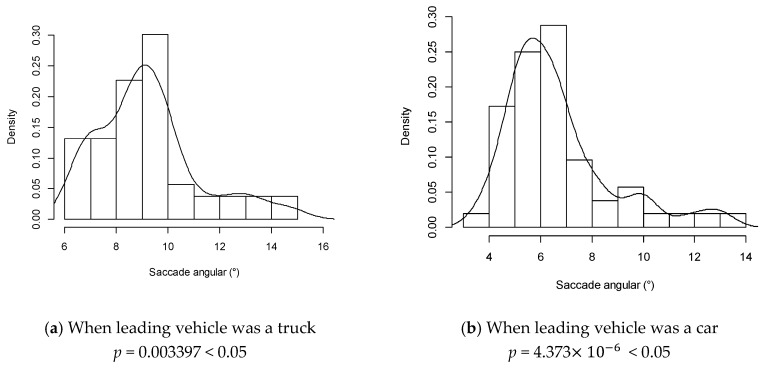

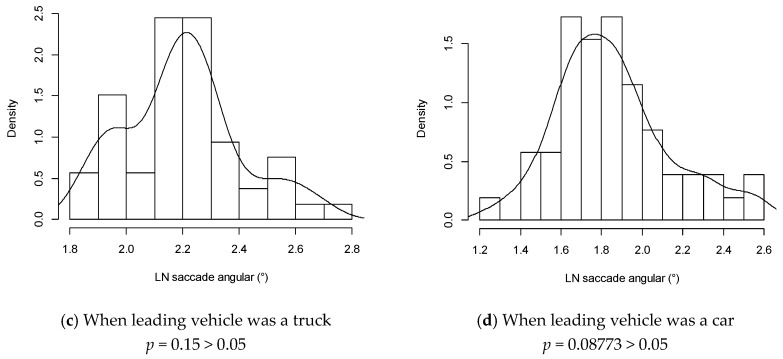
Distribution of a subject’s saccade angles.

Subjects’ saccade angles spread widely from 5° to 50°. Saccade angles at 60 km/h did not follow a normal distribution pattern, as shown in Figure 4a,b. After log-normal (LN) transformation of the saccade angles, it roughly followed a normal distribution pattern, as shown in Figure 4c,d. The same conclusion could be drawn at other speed levels. The drivers’ saccade angle followed a log-normal distribution pattern during overtaking maneuvers accordingly.

As the speed of a leading vehicle increased, participants tended to have a wider saccade angle. ANOVA shows that the speed of the leading vehicle had a notable influence on the mean saccade angle (truck: *p* = 0.0217 < 0.05; car: *p* = 0.0292 < 0.05). However, the effect of the type of the leading vehicle on saccade angles was not significant. 

### 4.3. Visual Staying and Shifting Probability

Subjects keep shifting their viewpoints from one AOI to another in order to capture sufficient traffic information. Visual staying and shifting probability can reveal drivers’ visual searching modes. Visual shifting probability (*P_ij_*) is defined as the ratio of the shifting times between the area *i* and *j* to total times.

(1) $$\begin{array}{ccc} P_{ij}=\frac{n_{ij}}{\sum_{i=1}^{5}\sum_{j=1}^{5}n_{ij}} & & \left(i\neq j\right) \end{array}$$

(2) $$\sum_{i=1}^{5}\sum_{j=1}^{5}P_{ij}=1\;\left(i\neq j\right)$$

*i* or *j* represents the AOI, *i*, *j* = 1,2,⋯,5. *n*_ij_ represents the shift times from the area *i* to *j*.

The visual stay duration equals the gaze duration plus saccade duration. When i=j, *P_i_* is the visual stay probability in an AOI.

(3) $$T_{i}=G_{i}+S_{i}\;\left(i=j\right)$$

(4) $$P_{i}=\frac{T_{i}}{\sum_{i=1}^{5}T_{i}}\;\left(i=j\right)$$

(5) $$\sum_{i=1}^{5}P_{i}=1\;\left(i=j\right)$$

$T_{i}$ represents the visual staying duration in area *i*. $G_{i}$ and $S_{i}$ represents the gaze and saccade duration, respectively.

BeGaze was used to output the values of parameters in the Equations (1)–(5). Subjects’ visual shifting and staying probabilities between AOI during overtaking are shown in Table 4.

As seen from Table 4, if i=j, the maximum visual staying probability is $P_{33}$ or $P_{44}$, and the sum of $P_{33}$ and $P_{44}$ accounts for more than 65% ($\text{max}\left\{P_{ij}/i=j\right\}=P_{33}\;or\;P_{44}$, $P_{33}$ + $P_{44}$ > 65%). The visual staying probabilities in LM, RM, and OA ($P_{11}$, $P_{22}$, and $P_{55}$) accounted for about 7%, 7%, and 10%, respectively. The visual staying probability in either the IL or DL were both significantly higher. Subjects tended to pay more attention to IL and DL during overtaking maneuvers. When subjects overtook a truck at a higher speed (80 km/h or more), their visual staying probability in the IL was a little higher than in the DL ($P_{44}>P_{33}$). At lower than 80km/h speed, however, $P_{33}$ was higher than $P_{44}$. When subjects overtook a car, the visual staying probability in the IL was higher than in the DL at any speed level ($P_{44}>P_{33}$).

If i≠j, the visual shifting probability from DL to IL or from IL to DL was higher ($\text{max}\left\{P_{ij}/i\neq j\right\}=P_{34}\;or\;P_{43}$), accounting for about 20%. From here we can see that subjects more frequently shifted their fixations between the IL and DL during overtaking maneuvers.

Furthermore, we can see that:

(6) $$\begin{array}{l} \text{max}\left\{P_{i1}/i\neq 1\right\}=P_{31}\;\text{or}\;P_{41}\\ \text{max}\left\{P_{i2}/i\neq 2\right\}=P_{32}\;\text{or}\;P_{42}\\ \text{max}\left\{P_{i5}/i\neq 5\right\}=P_{45}\\ \text{max}\left\{P_{1j}/j\neq 1\right\}=P_{13}\;\text{or}\;P_{14}\\ \text{max}\left\{P_{2j}/j\neq 2\right\}=P_{23}\;\text{or}\;P_{24}\\ \text{max}\left\{P_{5j}/j\neq 5\right\}=P_{54} \end{array}$$

Therefore, when subjects scanned the wing mirrors, the previous visual point and the next visual point more likely fell into the IL or DL. Moreover, when subjects scanned an OA, the previous visual point and the next visual point tended to fall into the IL. $P_{12}\;\text{and}\;P_{21}$ exhibit relatively lower probabilities, which indicates that subjects seldom shifted their visual points between the left mirror and the right mirror directly.

## 5. Discussion

### 5.1. Gaze Behavior

In general, the type of the leading vehicle influenced the gazing behavior. Participants had longer gaze duration and lower gaze frequency when a truck served as the leading vehicle. One possible explanation for this result is that the profile of a vehicle is one of the sensitive factors for a crash [51]. The larger the size of the vehicle, the higher the risk it may bring. While overtaking a truck, drivers tended to give more careful observations on average because of the larger space occupied by the body of the truck. Drivers may operate more nervously while changing lanes and accelerating, thus their gaze duration will be longer. Some psychology experiments also indicate that the gaze duration will extend when the subjects focus on an object [52]. However, the participants tended to feel more at ease when overtaking a car. As a car has a smaller profile, participants may have found it easier to observe sufficient traffic information. As such, they might not have needed to take a longer time to fix their viewpoints on a position.

The present findings appear to be consistent with the research presented by Panos Konstantopoulos, Peter Chapman, and David Crundall [38] which indicated that more hazards resulted in longer fixations and lower sampling rates. This is considered crucial in hazardous situations when drivers need longer time to process information. These findings may provide helpful guidance for novice drivers when they initiate overtaking maneuvers. 

### 5.2. Saccade Behavior

**Figure 5:**
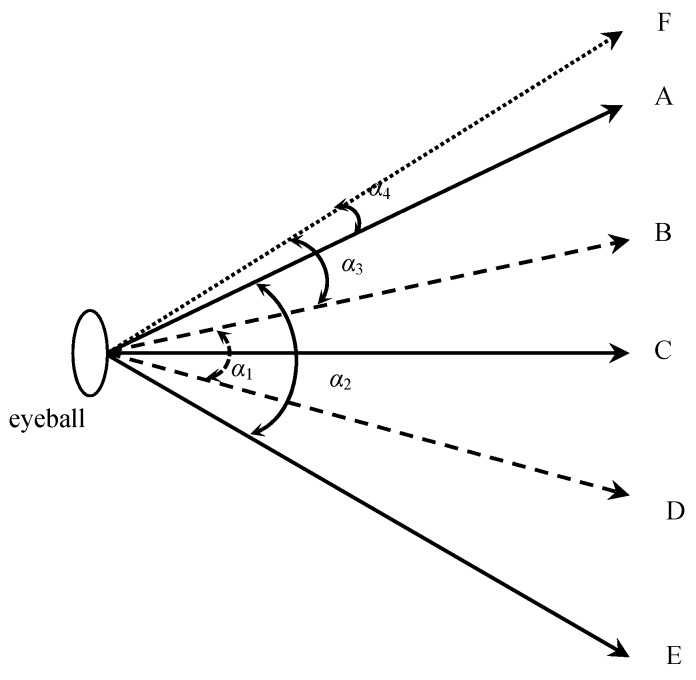
Visual fields of drivers. The scope between direction A and E represents the visual angle at a lower speed. The scope between direction B and D represents the visual angle at a higher speed. Direction C shows the horizontal direction of drivers’ eyeballs. Expected traffic information lies in the scope between direction B and F or between direction A and F.

The speed of the leading vehicle influenced the saccade duration and angle. The higher the speed, the longer the saccade duration and the larger the saccade angle tended to be. A possible explanation is that drivers’ visual fields become narrow as vehicles’ speeds increase [53]. As is shown in Figure 5, *α*_1_ is the visual field at a higher speed, and *α*_2_ corresponds to the visual field at a lower speed, with *α*_1_ < *α*_2_. If drivers want to catch the traffic information at the area F, their saccade angles will thus be *α*_3_ at a higher speed and *α*_4_ at a lower speed (with *α*_3_ > *α*_4_). During overtaking maneuvers, drivers continuously gathered information from the five AOI. The mean saccade angle and saccade duration increased with the speed of the leading vehicle increasing accordingly. According to the present findings, some auxiliary safety devices may be developed to supplement information for drivers (e.g., a horizontal and lateral safety distance warning system and video image technology) [54,55].

### 5.3. Visual Staying and Shifting 

During overtaking maneuvers, participants should pay more attention to the traffic information on the lanes, especially the distance with the leading vehicle or other vehicles. As a result, both the IL and DL are the key areas which participants focus on. While driving under normal circumstances on a freeway, drivers typically tend to allocate their viewpoints more widely. For example, the instrument panel, outside of the road, and other traffic signs may also attract drivers’ attentions. Panos Konstantopoulos, Peter Chapman, and David Crundall [12] found that the stay probability in the left or right mirror was about 5%, a little lower than our results. In their research, which differed from our own experiments, subjects drove on the simulating urban road freely, thus, they spread their viewpoints more widely. 

As participants allocate their viewpoints more on the IL and DL, the visual shifting probabilities between these two regions are naturally higher. The results provide good guidance for drivers on how to allocate their viewpoints and reduce collision risks during overtaking maneuvers. 

## 6. Conclusions

Overtaking is among the most demanding maneuvers on a freeway, requiring both driving and visual skills. Although there have been some achievements on safe distance modeling and driving operations, limited research focuses on drivers’ visual search modes. The present study examined the data of drivers’ eye movements extracted from a driving simulator and displays the appropriate visual allocating modes.

The findings in this study show that the type of leading vehicle influences the gaze behavior of participants, and the speed of a leading vehicle influences saccade behavior. This work may facilitate the traffic accident analyses related to overtaking maneuvers, and also is of great help for the drivers training based on visual behavior patterns. The key visual areas and visual shifting pattern between AOIs may provide beneficial guidance for drivers during overtaking. However, drivers’ operation is not taken into consideration in this study. To obtain the correlation between driving behavior and eye movements, focused study on the operating data, such as steering and accelerating during overtaking, will be conducted in future work.

## Figures and Tables

**Table 1:** Descriptive statistics of the participants’ sample data.

Statistics	Min	Max	Mean	Standard Deviation
Age	29	54	37.25	14.63
Years of licensed driving experience	2	22	10.84	22.56
km traveled	21,000	1,200,000	110,126.45	121,225.57

**Table 2:** Gaze duration and frequency during overtaking.

Gaze Behavior	Leading Vehicle	Speed (km/h)				
		60	70	80	90	100
Mean gaze duration (ms)	Truck	626.53	608.18	582.69	575.62	579.61
	Car	541.54	523.21	520.54	516.97	516.25
Gaze frequency (Hz)	Truck	1.14	1.22	1.26	1.24	1.21
	Car	1.40	1.43	1.42	1.35	1.37

**Table 3:** Saccade behavior during overtaking.

Saccade Behavior	Leading Vehicle	Speed (km/h)				
		60	70	80	90	100
Mean saccade duration (ms)	Truck	46.21	55.92	58.55	65.89	102.51
	Car	45.24	45.99	51.07	59.22	97.69
Saccade frequency (Hz)	Truck	1.02	1.04	1.09	1.18	1.04
	Car	1.15	1.27	1.22	1.17	1.24
Mean saccade angle (°)	Truck	9.14	13.64	14.77	21.24	39.63
	Car	6.36	8.69	13.61	17.28	33.61

**Table 4:** Visual staying and shifting probabilities between AOI.

Speed (km/h)	Type of a Leading Vehicle										
	Truck						Car				
	AOI	RM (1)	LM (2)	DL (3)	IL (4)	OA (5)	RM (1)	LM (2)	DL (3)	IL (4)	OA (5)
60	RM (1)	0.0722	0.0000	0.0660	0.0328	0.0035	0.0855	0.0000	0.0555	0.0744	0.0000
	LM (2)	0.0000	0.0612	0.0306	0.0668	0.0010	0.0000	0.0602	0.0343	0.0647	0.0031
	DL (3)	0.0663	0.0683	0.3374	0.2084	0.0278	0.0678	0.0608	0.3519	0.2461	0.0060
	IL (4)	0.0368	0.0473	0.1747	0.4520	0.0595	0.0591	0.0375	0.2193	0.4256	0.0216
	OA (5)	0.0015	0.0057	0.0486	0.0546	0.0722	0.0029	0.0000	0.0191	0.0277	0.0768
70	RM (1)	0.0748	0.0028	0.0566	0.0644	0.0028	0.0769	0.0000	0.0463	0.0612	0.0000
	LM (2)	0.0000	0.0692	0.0371	0.0889	0.0018	0.0000	0.0768	0.0535	0.0615	0.0028
	DL (3)	0.0489	0.0602	0.3524	0.1715	0.0183	0.0580	0.0734	0.3457	0.2088	0.0127
	IL (4)	0.0749	0.0614	0.1816	0.3973	0.0447	0.0420	0.0355	0.2004	0.4060	0.0567
	OA (5)	0.0046	0.0062	0.0328	0.0406	0.1063	0.0024	0.0106	0.0211	0.0531	0.0946
80	RM (1)	0.0730	0.0049	0.0689	0.0542	0.0000	0.0813	0.0000	0.0646	0.0566	0.0000
	LM (2)	0.0111	0.0858	0.0464	0.0690	0.0011	0.0000	0.0674	0.0276	0.0767	0.0000
	DL (3)	0.0670	0.0735	0.4088	0.1919	0.0142	0.0757	0.0650	0.3626	0.2066	0.0157
	IL (4)	0.0539	0.0412	0.1611	0.3411	0.0575	0.0372	0.0475	0.1925	0.3898	0.0520
	OA (5)	0.0000	0.0095	0.0302	0.0443	0.0913	0.0028	0.0046	0.0218	0.0532	0.0989
90	RM (1)	0.0621	0.0072	0.0397	0.0467	0.0076	0.0806	0.0000	0.0295	0.0607	0.0037
	LM (2)	0.0049	0.0790	0.0289	0.0757	0.0075	0.0000	0.0819	0.0435	0.0946	0.0040
	DL (3)	0.0531	0.0603	0.3748	0.2200	0.0328	0.0193	0.0902	0.3082	0.2114	0.0275
	IL (4)	0.0382	0.0428	0.2065	0.3374	0.0336	0.0740	0.0316	0.1982	0.3499	0.0351
	OA (5)	0.0051	0.0067	0.0432	0.0403	0.1467	0.0016	0.0040	0.0317	0.0395	0.1794
100	RM (1)	0.0742	0.0111	0.0524	0.0506	0.0063	0.0724	0.0000	0.0349	0.0584	0.0063
	LM (2)	0.0114	0.0777	0.0472	0.0923	0.0056	0.0063	0.0677	0.0379	0.0559	0.0072
	DL (3)	0.0355	0.0810	0.4063	0.1708	0.0319	0.0528	0.0487	0.3285	0.1915	0.0224
	IL (4)	0.0600	0.0456	0.1663	0.3274	0.0386	0.0745	0.0439	0.2101	0.4258	0.0536
	OA (5)	0.0100	0.0056	0.0299	0.0480	0.1144	0.0054	0.0084	0.0269	0.0547	0.1056

RM, right mirror; LM, left mirror; DL, destination lane; IL, initial lane; OA, other area.

## Abbreviations

The following abbreviations are used in this manuscript:

HED	SMI iView X HED 4 system
BeGaze	Behavioral and Gaze Analysis 2.5 system
AOI	area of interests
RM	right mirror
LM	left mirror
DL	destination lane
IL	initial lane
OA	other area
